# A Proposed Role for Pro-Inflammatory Cytokines in Damaging Behavior in Pigs

**DOI:** 10.3389/fvets.2020.00646

**Published:** 2020-10-02

**Authors:** Janicke Nordgreen, Sandra A. Edwards, Laura Ann Boyle, J. Elizabeth Bolhuis, Christina Veit, Amin Sayyari, Daniela E. Marin, Ivan Dimitrov, Andrew M. Janczak, Anna Valros

**Affiliations:** ^1^Department of Paraclinical Sciences, Faculty of Veterinary Medicine, Norwegian University of Life Sciences, Oslo, Norway; ^2^School of Natural and Environmental Sciences, Newcastle University, Newcastle upon Tyne, United Kingdom; ^3^Teagasc Animal and Grassland Research and Innovation Centre, Fermoy, Ireland; ^4^Adaptation Physiology Group, Wageningen University & Research, Wageningen, Netherlands; ^5^Department of Production Animal Clinical Science, Faculty of Veterinary Medicine, Norwegian University of Life Sciences, Oslo, Norway; ^6^National Institute for Research and Development for Biology and Animal Nutrition, Balotesti, Romania; ^7^Agricultural Institute, Stara Zagora, Bulgaria; ^8^Department of Production Animal Medicine, Research Centre for Animal Welfare, University of Helsinki, Helsinki, Finland

**Keywords:** cytokines, pig, social behavior, tail biting, health–clinical

## Abstract

Sickness can change our mood for the worse, leaving us sad, lethargic, grumpy and less socially inclined. This mood change is part of a set of behavioral symptoms called sickness behavior and has features in common with core symptoms of depression. Therefore, the physiological changes induced by immune activation, for example following infection, are in the spotlight for explaining mechanisms behind mental health challenges such as depression. While humans may take a day off and isolate themselves until they feel better, farm animals housed in groups have only limited possibilities for social withdrawal. We suggest that immune activation could be a major factor influencing social interactions in pigs, with outbreaks of damaging behavior such as tail biting as a possible result. The hypothesis presented here is that the effects of several known risk factors for tail biting are mediated by pro-inflammatory cytokines, proteins produced by the immune system, and their effect on neurotransmitter systems. We describe the background for and implications of this hypothesis.

## Introduction

The immune system of mammals is activated by pathogens or by non-infectious insult. Molecules from the pathogens or from damaged tissue will activate the immune system through receptors responding to pathogen or damage associated molecular patterns. The innate immune-system is the first to respond, followed by an antibody-dominated and/or cellular response from the adaptive immune system. Both the innate and the adaptive part of the immune system produce cytokines, small proteins that are central both in orchestrating the immune response and in protecting the body against further damage. Cytokines can be either pro- or anti-inflammatory, and the balance between these is important in avoiding an uncontrollable immune response. The pro-inflammatory cytokines that are best described in terms of effects on mood and behavior are interleukin 1 beta (IL-1β), interleukin 6 (IL-6) and tumor necrosis factor alpha (TNF-α) (1–5). These cytokines are produced during the early stage of the immune response and induce changes in behavior and physiology such as fever, anorexia, decreased thirst, lethargy, decreased social motivation, changed sleeping pattern, decreased grooming, anhedonia (6, 7), and anxiety (5). These changes, usually referred to as “sickness response,” are all highly adaptive (6), and represent a shift in motivational state that allows the individual to conserve energy and stay out of harm's way in order to recover. The behavioral changes are collectively called “sickness behavior,” and they share features with some types of mental illness, such as anxiety and depression (1, 6, 8, 9). This has led to the hypothesis that immune activation could be the culprit behind many cases of psychological disease in humans, and has given a new dimension to the mood deterioration experienced by patients with cardiac disease, metabolic syndrome and chronic fatigue syndrome (10–12). A high percentage of patients undergoing immune therapy for hepatitis or metastatic cancer experience severe psychological side effects (13–15), lending support to cytokines as a causal factor in mood deterioration.

The aim of this paper is to discuss the hypothesis that cytokines mediate the effect of different environmental stressors on individual pigs to increase the likelihood of the pig showing damaging behavior, such as tail biting or ear biting, toward other members of its social group. Tail biting, the damaging biting of the tail of another pig, is one of the gravest problems in intensive pig production. Many risk factors for tail biting are well described, such as suboptimal nutrition, poor climatic conditions, and lack of rooting materials (16). Less is known about ear biting, but this is also a damaging behavior and associated with tail biting (17). Tail and ear biting are believed to have a different motivational background than aggressive biting and fighting, which is normally directed toward the front and sides of the body of the recipient (18) The mechanisms through which the risk factors work are not known, and tail and ear biting are notoriously difficult to induce experimentally, making research on causal relationships hard. In this paper, we draw upon literature from human and rodent studies as most of our knowledge of the effect of immune activation on mood and behavior originate in those species. Pigs, mice, and human all show sickness behavior, and there are considerable similarities between the pig and human immune system (19). The features of the immune response that are central to the hypothesis presented in this paper are shared by pigs, humans, and mice. Those key features will be described in detail in the following paragraphs, but can be summarized as a production of pro-inflammatory cytokines in response to immune stimulation, and a following shift in tryptophan metabolism.

Sickness may influence the likelihood of damaging behavior in different ways. A pig weakened through illness may be perceived as less competitive by its penmates, and would have more difficulty escaping bullying. However, an increased likelihood of victimization in sick pigs does not contribute to explaining what causes a biter to bite and, as mentioned, it is the latter question that will be addressed in this paper. The hypothesis that cytokines may be central agents in the development of damaging behavior rests on the two following lines of reasoning: Firstly, many stressors, not only pathogens or physical damage, can elicit an immune response. Several of the known risk-factors for damaging behavior in pigs stimulate the immune system, and this will be discussed in the sections Pathogens and immune activation, The housing environment and immune activation, Psychological stress and immune activation and Mycotoxins in feed. Secondly, while lethargy and withdrawal do not seem like states that would induce damaging behavior, they are not the only behavioral changes brought about by immune activation. Irritability and short temper, extreme emotional lability, tearfulness and cognitive impairment are reported in human clinical studies on the effects of treatment with pro-inflammatory cytokines such as IL-2 and interferon α (IFNα) (13–15, 20). Anxiety can also be induced by treatment with IL-1β (5), and increased fearfulness has been described in tail biting pigs (21). There are indications that inflammatory proteins may play a role in aggression as elevated levels of IL-6 and C-reactive protein (CRP) were found in psychiatric patients with a diagnosis of intermittent explosive disorder, and patients on IFN-α therapy had increased hostility/aggression scores (22–24). Irritability is listed as a core symptom of depression in children and adolescents in DSM-IV (the diagnostic and statistical manual of mental disorders) and there are discussions as to its importance and prevalence also in other age groups [discussed in (25–27)]. In pigs, increased irritability may, together with lower social motivation, explain a lower threshold for showing damaging behavior toward penmates in pigs with a pro-inflammatory immune response.

In this paper we will discuss the effects of cytokines on brain neurotransmitter systems, present the literature on how four putative risk factors for tail biting (disease, non-hygienic barn environment, psychological stress, and mycotoxins) influence the level of cytokines and how the composition of feed can influence both neurotransmitter balance and the response to immune activation, and link this to the suggested role of the gut microbiota in damaging behavior (28). Lastly, we will discuss the implication of our hypothesis for the keeping of pigs in intensive production systems and suggest directions for future research into how the immune system influences behavior.

## The Effects of Cytokines on Brain Physiology and Function

Cytokines are proteins produced by several types of immune competent cells. They have various roles in the immune response, and orchestrate both the physiological and behavioral changes needed to combat challenges to the organism. An increase in cytokine production in the periphery may influence the brain by vagal stimulation, and cytokines can influence the brain directly either (probably) by volume diffusion after production in the circumventricular organs, by binding to IL-1 receptors in brain venules, through cytokine transporters across the blood brain barrier, by synthesis in the blood endothelial cells or by synthesis in the CNS by glial cells (1, 28–32).

**Figure 1:**
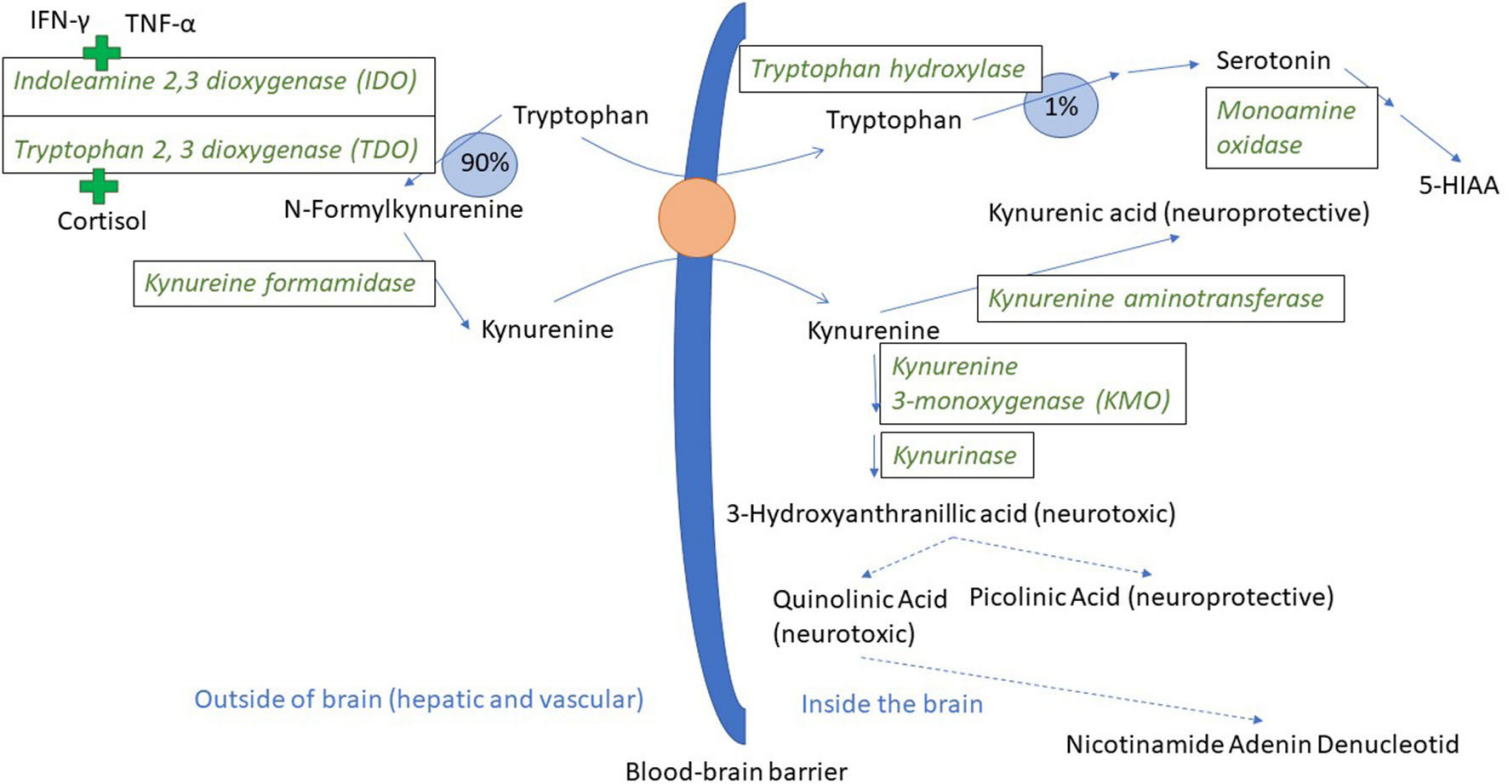
Tryptophan and kynurenine metabolism and transport across the blood-brain barrier. In a normal physiological state, ~90% of the tryptophan that is not incorporated into protein is metabolized through the kynurenine pathway (44). Tryptophan and kynurenine both access brain tissue through the large neutral amino acid transporter protein here visualized as an orange circle (45). The green plus signs indicate the induction of IDO and TDO by pro-inflammatory cytokines and cortisol, respectively. 5-HIAA is 5-hydroxyindoleacetic acid. Broken arrows indicate metabolic steps not detailed in the figure.

The innate immune system is the first line of defense against disease and non-infectious damage. Dendritic cells and macrophages will produce the three major pro-inflammatory cytokines IL-1β, TNF-α, and IL-6 during the early stages of inflammation. These three cytokines are the most extensively studied in terms of effects on mental health, and agents such as LPS (lipopolysaccharide, a component of the cell wall of gram negative bacteria), that induce an innate immune response, are used to model inflammatory-induced depression and also anxiety in rodents (33–35). IL-1β, TNF-α, and IL-6 stimulate acute phase protein production in hepatocytes, and prostaglandin E2 production in the CNS, inducing a fever response (36, 37). TNF-α also leads to increased synthesis of the enzyme indoleamine 2, 3 deoxygenase 1 (IDO-1, hereafter called IDO) (38, 39). IDO can be expressed in endothelium, macrophages and dendritic cells in all organs of the body including the brain [(40) (mouse), (41) (mouse), (42) (mouse)], and catabolises tryptophan to kynurenine (43). Tryptophan is an essential amino acid. Under normal physiological conditions, tryptophan is the precursor both for the neurotransmitter serotonin and for kynurenine (Figure 1). As part of the inflammatory response, IDO is upregulated and relatively more tryptophan goes into kynurenine synthesis rather than serotonin synthesis (33, 46, 47). Interferon-γ, a cytokine produced by natural killer (NK) cells both during the innate and adaptive immune response, is also a powerful inducer of IDO (48). The adaptive function of tryptophan depletion that is partly brought about by increased IDO activity is to reduce tryptophan availability for infectious agents (49). Depletion of tryptophan in plasma was demonstrated in pigs following experimentally induced lung inflammation (50) and a chronic immunological challenge induced by unhygienic housing conditions (51), making the processes described below relevant for naturally occurring immune stimulation. Pigs injected with LPS also show a reduction in plasma tryptophan and an increase in kynurenine in the first hours following injection (52).

As mentioned, IDO can be found in most body tissues, including the brain. However, the majority of kynurenine in the brain comes from the blood (53). Kynurenine relies on the same transport protein as tryptophan (45), and can thus compete with tryptophan for access to the brain. When in the brain, kynurenine is broken down either to kynurenic acid or to quinolinic and picolinic acid via the intermediary 3 hydroxy kynurenine (Figure 1). Kynurenic acid is mainly produced by astrocytes and is an *antagonist* on the NMDA (N-methyl-D-aspartate) type of glutamate receptor, and has neuroprotective effects. Quinolinic acid is produced by microglia and is an NMDA receptor *agonist* (54, 55). Kynurenic acid will reduce glutamate release in the brain, whereas quinolinic acid can have the opposite effect. Ketamine, an NMDA receptor antagonist, blocked LPS-induced signs of depression in mice, lending support to the hypothesis that NMDA receptor activation might be important for the psychological sequela to acute inflammation (47). NMDA receptor antagonists are now under intense study as novel rapidly acting antidepressants (56). Noradrenaline is also implicated as important in the development of psychiatric disease and can influence the expression of different subcomponents of glutamate receptors (57). Mice injected with LPS had a significant drop in frontal cortex noradrenaline levels 24 h after injection, and fluoxetine (a selective serotonin reuptake inhibitor) decreased this effect and the depression-like effects as well (58). Pigs injected with LPS had significantly lower noradrenaline levels in their hypothalamus, hippocampus, and frontal cortex 72 h after LPS injection (52).

Thus, increased IDO activity can lead to two changes in the balance of neurotransmittors/neuromodulators in the CNS: it can decrease the level of serotonin and increase the level of kynurenine and downstream metabolites, all through the shunting of tryptophan from serotonin and into kynurenine synthesis. Serotonin is an important neurotransmitter, and long implicated in the etiology of depression (59), and kynurenine and downstream metabolites can influence both neurons and glia as summarized above. Therefore, both of these processes may lead to the depressive changes seen in mice injected with LPS. Whether the serotonin reduction, the kynurenine increase or both are most important is still a matter of debate. Some of the evidence for both serotonin and kynurenine and downstream metabolites as causal agents in the development of depression is presented below, and in the last paragraph, related to the literature linking the serotonergic system to tail biting in pigs.

Several studies and meta-analyses show a correlation between depression (in human subjects) and decreased plasma tryptophan levels, as well as a link between immune therapy and decreased plasma tryptophan (60–62). Fewer experiments have been designed in a way that supports a clear causal link between tryptophan levels and/or brain serotonin levels and mood [discussed in (1)]. One study indicating causality showed that experimental tryptophan depletion in humans lowered mood in individuals with a history of depression who were in remission and under treatment with antidepressant drugs (63). Mice injected with bacilli Calmette-Guerin had increased IDO activity in their brain tissue, a drop in serum tryptophan but no significant changes in brain tryptophan levels (64). In an LPS model of inflammation-induced depression in mice, the LPS group showed changes in behavior interpreted as depression-like, an increase in both serotonin levels and serotonin turnover alongside an increase in IDO expression and kynurenine/tryptophan ratio in brain tissue. The depression-like changes in behavior were blocked by treatments inhibiting IDO activity directly or indirectly, but without any influence on the LPS-induced increase in serotonin turnover (33). This indicates that at least with the experimental set-up described by O'Connor et al. the product of IDO, kynurenine, and/or metabolites further downstream may be more important mediators of the depressive-like changes than a decrease in serotonin levels, and that tryptophan availability was sufficient for serotonin synthesis, also during inflammation. Interestingly, the tryptophan levels in plasma was reduced 28 h after LPS injection, but the brain level of tryptophan was slightly increased, showing a lack of correlation between blood and brain tryptophan levels similar to that reported by Moreau et al. (64). Thus, looking at the evidence from different species and models together, the literature on the link between inflammation, IDO-activation, tryptophan, and serotonin levels and mood is not conclusive when it comes to the importance of serotonin in the depressive-like behavioral changes. Importantly, the sampling area, and the time of sampling relative to immune stimulation as well as availability of tryptophan through the diet might influence the results, and a study focusing on the mouse frontal cortex did find lower serotonin and noradrenaline levels 24 h after LPS injection compared to controls (58).

Tryptophan supplementation in pigs has decreased ear biting and tail biting (65), and modified the effect of being housed in a suboptimal environment (66), showing that tryptophan can influence behavior. Interestingly, pigs have the ability to detect dietary deficiencies (67), such as a deficiency in tryptophan (68). This is discussed in more detail in the paragraph amino acid composition of feed. A few studies have looked into a possible association between the functioning of the serotonergic system and damaging behavior. Pigs performing tail biting behavior have lower levels of serotonin stored in blood platelets, and higher serotonin uptake velocity into the blood platelets (21). The serotonin transporter in blood platelets is used as a peripheral marker of CNS serotonin transporter activity (69), and the results indicate that the serotonin transport proteins of tail biters could function differently from serotonin transport proteins in control (neither victim nor biter) pigs. The correlation between blood platelet serotonin storage and hippocampal serotonin levels was negative and the correlation with serotonin turnover was earlier found to be positive (70). There was also a negative correlation between uptake velocity and 5-HIAA (5-hydroxyindoleacetic acid; the main serotonin metabolite) in the frontal cortex. Based on these reports, a tail biting pig would be expected to have lower levels of serotonin turnover and higher serotonin levels in their hippocampus, but lower 5-HIAA levels in the frontal cortex. However, Valros et al. (71) found a tendency to increased 5-HIAA levels in the prefrontal cortex of tail biters compared to controls, but no other differences in brain serotonin levels or turnover between victims, biters, and controls. It is worth noting that tail biters alone showed a correlation between the ratio of tryptophan to other amino acids, and to 5-HIAA and serotonin in the frontal cortex (71). The latter finding could indicate a stronger dependency of serotonin levels on tryptophan availability in tail biters than in other pigs.

In summary, pro-inflammatory cytokines like TNF-α and IFN-γ will increase the expression of IDO, which will in its turn increase the production of kynurenine from tryptophan. Whether this reduces serotonin levels is not completely clear and might depend on tryptophan availability from the diet (see section Amino acid composition of feed). Kynurenine metabolites have both NMDA agonistic and antagonistic effects and the IDO-led shunt toward kynurenine seems to be important in the development of psychological symptoms following immune activation. However, the steps leading from pro-inflammatory cytokines to a long-term change in subjective experience as well as behavior are still incompletely understood. One challenge is to move away from single biomarkers toward a coherent picture of the neurophysiological changes underlying both short and long-term mood changes; another is to move from detecting correlations to testing causality. Lastly, most of the literature is concerned with the link between immune activation and feelings of helplessness, social withdrawal and anhedonia. Less is known about whether irritability and aggression are influenced by the same pathways.

## Risk Factors for Tail Biting in Pigs and Their Relationship With Cytokines

A high number of housing and management-related risk factors for tail biting in pigs have been identified, illustrated e.g., by the diversity in the on-farm tail biting husbandry advisory tool HAT, which includes 83 risk factors (16). Some of the most commonly mentioned risk factors include feeding-related problems (72, 73), poor environmental conditions, such as suboptimal temperature, draft, and poor air quality (72–74), lack of enrichment (72, 75), suboptimal diet composition (76, 77), high animal density, (74, 78), slatted flooring (79, 80), and reduced health status (79–82). Stress induced by the above listed risk factors might be one of the explanations for the suggested effects on damaging behavior, as increased stress levels are connected to an increased risk of tail biting (83, 84).

In this section, the relationship between the risk factors pathogens (Pathogens and immune activation), an unsanitary barn environment (The housing environment and immune activation), psychological stress (Psychological stress and immune activation), mycotoxins (Mycotoxins in feed), and immune activation will be discussed. The influence of diet composition on the effects of cytokines on brain neurochemistry (Amino acid composition of feed) highlights the importance of understanding the physiological background upon which risk factors exert their effects in order to better predict the behavioral outcome.

### Pathogens and Immune Activation

Based on epidemiological studies, case control studies and clinical reports, poor health due to infection with pathogens is considered a risk factor for tail biting (reviewed in Boyle et al. in preparation).

The inflammatory response will differ depending on what the animal is exposed to, be it viruses, intracellular bacteria or extracellular bacteria, or non-infectious damage. A viral infection will in general stimulate synthesis of interferons α and β, while bacterial infection initiates the IL1-β, TNF-α, IL-6 chain of events. However, in both cases NK-cells will be activated to produce IFN-γ. The major pro-inflammatory cytokines can be produced by T-cells in later stages of viral infection, or by macrophages responding to damage- or pathogen- associated molecular patterns. Thus, most of the inducers of the inflammatory response will lead to production of cytokines (IFN-γ and TNF-α) that can activate IDO and change tryptophan metabolism, thereby reducing tryptophan levels in plasma. Cytokine responses were described in pigs after both experimental challenges and naturally occurring disease, and examples are given in Table 1 for some common respiratory and gastrointestinal diseases.

**Table 1:** Agent, cytokine, tissue, and species is shown for some common respiratory and gastrointestinal disease in pigs.

**Respiratory disease**			
**Infectious agent**	**Cytokine response**	**Tissue**	**References**
*Actinobacillus pleuropneumoniae*	Increase: IL-6, IL-1α, TNF-α	Serum (pig)	(85)
*Mycoplasma hyopneumoniae*	Increase: IL-1α and -β,TNF-α, IL-6, IL-8, IL-5, IL-13, and IFN-γ	Airway and lung tissue (pig)	(86–88)
	Increase: IL-1β, IL-2, IL-4, IL-6, IL-8, IL-10, and TNF-α	Lymphatic tissue (pig)	(89, 90)
	Increase: IL-2, IL-4, TNF-α, IL-1α, and β and IL-6	Bronchoalveolar lavage fluid (natural infection) (pig)	(91)
*Influenza virus*	Increase: IL-8, IL-10, and IFN-γ	Lung (pig)	(92)
	Increase: IFN-γ, IL-1ra, and IL-4	Plasma (human)	(93)
*Porcine reproductive and respiratory syndrome virus*	Increase: IL-10, IFN-α, IFN-γ, IL-12p40, and TNF-α	Lung macrophages (pig)	(94)
**Gastrointestinal disease**			
**Infectious agent**	**Cytokine response**	**Tissue**	**Reference**
*Lawsonia intracellularis*	IL-8 and TNF-α	Enterocyte-derived porcine cell line	(95)
	TNF- α, IL-6, IFN-γ	Blood (pig)	(96)
*Escherichia coli*	IFN-γ, TNF-α, and IL-6	Blood (pig)	(97)
*Porcine circovirus type 2 (Postweaning Multisystemic Wasting Syndrome)*	IL-10 (monocytic cells *in vitro*)		(98)
	IL-10	Blood (pig)	(99)
	Increase: IFNy and IL8, slightly increased IFN-β, IL-1β and IL-12 Decrease: IFN-α and IL-4	Tracheobronchial lymph nodes (pig)	(90)
	IFN-α, IL-6, and IL-10	Serum (pig)	(100)
Weaning	IL-1β, IL-6, and TNF-α	Intestine (piglet)	(101)

*The effect on cytokine levels is an increase unless otherwise mentioned*.

In summary, *Actinobacillus pleuropneumoniae* (APP), *Mycoplasma hyopneumoniae, influenza virus*, and *porcine reproductive and respiratory syndrome virus* (PRRSv) are four important causal agents for respiratory disease in pigs (102). All of them will lead to a change in cytokine production, including an increase in TNF-α and IFN-γ which will lead to an increase in IDO activity.

Pigs are also prone to gastrointestinal disease, with *Lawsonia intracellularis, Escherichia coli, circovirus type 2*(PCV2) as three important infectious agents (102). Weaning-associated enteric disease is not that clearly linked to one agent, and it also includes psychological aspects (separation from dam, regrouping, novel environment), and will also be discussed under section Psychological stress and immune activation. The inflammatory response it induces is included in Table 1. *Lawsonia intracellularis* causes porcine proliferative enteropathy (PE). *Escherichia coli* is a gram-negative bacterium and LPS is an important component of the cell-wall (103). As discussed previously, LPS will activate the innate immune system. *Porcine circovirus type 2 (PCV2)* is associated with post-weaning multisystemic wasting syndrome (PMWS), and may also induce respiratory system symptoms and pathologies. Similar to the respiratory pathogens, all these gastrointestinal pathogens/conditions have the ability to increase TNF-α and/or IFN-γ, at least in some tissues, making them relevant in the discussion of the mental effects of IDO activation.

### The Housing Environment and Immune Activation

The pigs housing environment is characterized by factors such as illumination, noise, vibration, temperature and humidity, as well as gaseous and particulate matter (dust) in the air, derived from the animals, their effluent and feed. Behaviour there are significant costs to the animals associated with the continuous challenges posed by such environments (104, 105). For example, there is a higher frequency of pulmonary disease in commercial pig farms with poor hygiene (106).

Unhygienic environments are typically very dusty and exposure to dust triggers a cytokine response (107). Dust is not only a source of pathogens but is also composed of non-infectious particulate matter such as skin and feed particles and endotoxins released when bacterial cells die. Endotoxins causes respiratory problems in humans (108–110) as well as pigs (111, 112). Several authors reported neutrophil-mediated inflammation in naïve subjects and pig farm workers after even limited exposure to dust (113–115). It is thought that this is mediated by an acute inflammatory response involving a variety of cytokines, including the major pro-inflammatory cytokines IL-1, IL-6, IL-8, and tumor necrosis factor (TNF)-α, and subsequent massive recruitment and activation of neutrophils in the lower and upper airways (107). Both Van Reeth et al. (116) and Labarque et al. (117) demonstrated a synergistic effect of airborne bacterial LPS endotoxin on the severity of symptoms induced by the porcine reproductive and respiratory syndrome (PRRS) virus whereby development of PRRS with LPS exacerbated the production of pro-inflammatory cytokines, particularly TNF-a, in the lungs. Although less studied, non-endotoxin components of dust such as skin and feed particles also play a role in stimulating the immune response (118). For example, Sundblad et al. (119) observed that exposure to dust in the pig buildings was a much stronger pro-inflammatory stimulus than the inhalation of pure endotoxin (LPS), even though the doses of the LPS challenge were 200-fold higher than the doses inhaled in the farm.

The gaseous component of the air, in unhygienic environments, could also stimulate the immune response (104). For example, ammonia causes the release of cytokines by alveolar macrophages and neutrophils, constituting an inflammatory response (120). Exposure of zebrafish to high environmental ammonia activated the corticosteroid stress axis causing immunosuppression (121, 122). Similarly, in pigs, Von Borell et al. (123) reported higher serum cortisol levels in response to exposure to ammonia as well as increases in absolute monocyte, lymphocyte, and neutrophil counts and in haptoglobin concentrations. However, there was no response of the cytokine TNF-α to prolonged or acute exposure to ammonia in that study. Meanwhile, though Murphy et al. (104) found no direct effect of ammonia on the immune system, the combination of α-haemolytic cocci and ammonia stimulated an immune response in pigs.

In summary, unsanitary conditions with a high level of dust and gases such as ammonia can induce an inflammatory response very similar to the response launched after exposure to pathogens and could potentially affect brain physiology in much the same way.

### Psychological Stress and Immune Activation

Modern commercial pig rearing systems are known to potentially induce psychological stress in pigs, due to, among others, a lack of possibilities to perform highly motivated behaviors (124), or social stress caused by a high level of competition for resources, such as feed (125), or by regrouping (126). As stated previously, increased stress levels have been linked to tail biting and the immune response that can be induced by psychological stress could be one important mechanism behind this link.

In rodents, exposure to stressors are commonly used to model human depression-related physiological and behavioral changes. These models either induce psychological stress using short-term stressors, such as moving the animal to a novel environment (127), or chronic treatments, usually employing mild stress protocols over several days (128, 129). The latter aims to simulate long-term every-day negative stimuli, and is suggested to be a reliable model for depression (129, 130). In some studies, responses are further compared to an LPS-challenge, which is thought to mimic infection-induced human depression (127, 129). These studies showed that immune responses similar to those seen in humans experiencing stress (131) can also be induced in other species. Furthermore, psychological stress does appear to cause changes in cytokines and other immune parameters, which are similar to those seen as a response to LPS challenge (127, 129).

As an example, Maes et al. (131) showed that a prolonged period of stress (preparing for a demanding academic exam) caused an increase in serum IFN-γ, TNF-α, IL-6, IL-1ra, and IL-10-levels in medical students, as compared to cytokine concentrations measured more than a month before the exam or immediately afterwards. There were also significant correlations between the stress-induced changes in perceived stress, as self-rated by the students, and concentrations of IFN-γ, TNF-α, IL-6, and IL-1ra. In comparison, Zhao et al. (129) reported increased levels of serum and brain expression of TNF-α, IL-1β and IL-6 in mice subjected to a 4-week protocol of unpredictable chronic mild stress compared to control animals. These changes were generally comparable to, although milder than, those induced by LPS in the same study. Also using mice, Zhang et al. (128) showed that chronic mild stress for 4 weeks resulted in significantly higher levels of serum IL-1β and hippocampally active IL-1β. The above-mentioned studies modeled chronic stress, but an acute exposure to psychological stress, i.e., placement in a novel environment, also induced changes in central immune responses in rats in a manner that overlapped to some extent, but not completely with the pattern seen after immune activation by LPS injection (127). These authors found an increase in transcription factor NF-IL6, which is part of the molecular chain of events that leads to increased production of pro-inflammatory cytokines.

Behavioral changes characteristic of depression in rodents include, for example, altered responses in the open field, forced swim and tail suspension tests (128, 129), and decreased sucrose preference (128), all indicative of an increasingly passive state with signs of anhedonia (132). Correspondingly, in humans, depression includes signs such as enduring sadness, loss of interest or pleasure, decreased energy or feelings of tiredness, feelings of low self-worth, disturbed sleep or appetite, and poor concentration (133). However, as already mentioned in the introduction, also irritability and anger co-occur with other depressive symptoms in humans (134–136), and are suggested as clinical markers of a more severe, chronic, and complex depressive illness (135) which should receive more attention as the focus of future research (136).

In commercial pig production, the lack of environmental enrichment is commonly referred to as the most important risk factor for tail biting in pigs [reviewed by D'eath et al. (72)]. The suggested explanation is that pigs, which have a very high motivation to explore and manipulate their environment [reviewed by Studnitz et al. (137)], get bored and frustrated in a barren environment (138). Interestingly, environmental enrichment seems to also affect certain components of the immune system (139–141). In pigs, several authors reported effects of environmental enrichment on different aspects of the immune system and the acute phase response ((142–145)). None of these studies report specifically on cytokines, however, generally lower N:L ratios and haptoglobin concentrations in enriched-housed pigs could indicate that enriched-housed pigs are less stressed and has less immune activity which would be in line with the benefits of enrichment on pig behavior and welfare (146).

Another typical cause of psychological stress in pig production are social challenges due to, for example, regrouping and a high level of social competition. Recently, Gimsa et al. (147) reviewed multiple studies reporting a range of changes in immune functions in pigs due to psychosocial stress, caused by treatments such as social isolation, weaning and regrouping. In general, these authors concluded that chronic or repeated social stress protocols reduced immune responses and attenuated sickness symptoms. Very few of the studies included in this review, however, included central cytokine levels *per se*, however Kantiz et al. (148) and Tuchscherer et al. (149) reported an increased IL-1β level in the hippocampus due to repeated social isolation in young piglets. The socially stressed pigs in the study by Kanitz et al. (148) also showed more passive behavior than their non-isolated counterparts, which could be a sign of a depression-like state. Further, Tuchscherer et al. (150) found that the IL-6 mRNA expression in the hypothalamus was increased in pigs exposed to a single bout of isolation on postnatal day 7, and then challenged with *E.coli*, compared to non-isolated controls. They also found that the isolated piglets showed more clinical signs of sickness after the *E.coli* challenge, including behavioral changes, than non-isolated piglets.

In summary, psychological stress is known to induce production of pro-inflammatory cytokines such as IFN-γ and TNF-α both in humans and in rodent stress models. Pigs in commercial production systems can be subjected to psychological stress in several forms, and this seems to activate their immune system and lead to a stronger response to pathogen challenge. Thus, stressors can influence the immune system even when the insult is neither in the form of disease nor an unhygienic environment.

### Mycotoxins in Feed

Mycotoxins are secondary metabolites produced by different types of fungi (151). They are the most frequently occurring natural contaminants in both human and animal diets, and at least some of them are known to be able to induce a pro-inflammatory response. *Aspergillus, Fusarium*, and *Penicillium* are the main mycotoxin-producing fungi. Deoxynivalenol (DON) is one of the most prevalent *Fusarium* mycotoxins in grain and can represent an important health risk in animals [Reviewed in Payros et al. (152)]. The European Commission Recommendation 2006/576/EC has set the recommended maximum acceptable level for DON to 0.9 mg/kg, while the Norwegian national feed safety authority recommends a lower maximum acceptable level of 0.5 mg DON/kg for pig feed. Pigs are more sensitive to the CNS effects of DON exposure than mice and sheep (152). Acute exposure to high doses of DON in pigs may result in abdominal pain, increased salivation, diarrhea, and emesis (152, 153). However, under practical agricultural conditions, chronic exposure of pigs to moderate, rather than acute exposure to high doses of DON, constitutes the major concern. The most common chronic adverse health effects of DON in pigs are anorexia and consequently poor growth (154–156). Studies concerning the behavioral effects of mycotoxins, other than those related to anorexia, are scarce. Reduction in feed consumption and weight gain was attributed to the emetic effect of DON, possibly through the stimulation of the synthesis of pro-inflammatory cytokines as mediators of the acute phase reaction, and the capacity of DON to disrupt brain neurochemistry (157). Both of these hypotheses are relevant for the main synthesis of this review and will be described below.

One mg DON/kg body weight (by intravenous administration) increased the blood concentration of IL-8, TNF-α, and IL-6 in swine (158, 159). Also, administration of DON orally induced an up-regulation of IL-1β, IL-6, TNF-α, COX2, and microsomal prostaglandin synthase-1 mRNA within central structures involved in food intake control (160). The same authors (160) suggest that behavioral changes observed after DON intoxication differ from classical sickness behavior evoked by inflammatory cytokines, as the silencing of prostaglandin E2 signaling pathways did not modify the responses to the toxin. In addition to influencing cytokine production in the blood, DON can cross the blood-brain barrier with the potential of influencing neurons and glia directly (161, 162). A mapping of brain activation revealed that DON activated central structures such as hypothalamic nuclei and amygdala (159, 160). Faeste et al. (163) reported also that an acute high-DON (12.5 mg/kg bw) and chronic low-DON (100 μg DON/kg bw/ day) exposure in mice resulted in increased neuronal activity in the same brain regions and increased anxiety level in the chronic low-DON group. A complicating factor is that co-contamination of several mycotoxins seems to be prevalent, as described in a review of European multi-mycotoxin contamination studies (164). There are few published reports on the interaction between different mycotoxins and cytokine secretion. Bertrand et al. (165) reported that pigs fed diets contaminated with both DON and fumonisin B (FB) had a significant reduction in expression of several cytokines, including IL-1ß and IL-6, compared to control pigs. In another study, co-exposure to DON and FB resulted in an additive effect on the expression of TNF-α, an increased level of TNF-α when compared to a group treated only with DON, and a reduction in levels of IL-1ß and IL-6 compared with the group treated only with DON (166).

DON ingestion disrupts brain neurochemistry, and changes in both serotoninergic (167) and catecholaminergic (159) activities were reported after DON intoxication in swine. The effects of DON seem to be specific to each transmitter, time, and brain region (168, 169). Modifications of brain amine levels for dopamine, noradrenaline and serotonin were observed after intravenous DON administration (0.25 mg/kg) (167). We will focus on the changes in serotonin levels and -turnover in the following discussion. Serotonin content increased after 1 h in the hypothalamus but was reduced in the hypothalamus and the cortex after 8 h (168). Starter pigs fed grains naturally contaminated with *Fusarium* mycotoxins (DON, acetyl DON, and zearalenone) had increased serotonin turnover (measured as 5-HIAA/serotonin) in the hypothalamus and pons Swamy et al. (169), Swamy et al. (169). Al-Hazmi and Waggas (170) exposed mice to DON or FB and studied the effect of these mycotoxins on some brain catecholamines, serotonin and behavior. Mice that received mycotoxin had significantly higher concentrations of catecholamines in brain samples than control animals. In addition, this experiment showed an increase in the number of bites and aggressive behavior in animals intoxicated with DON or FB.

In summary, DON can induce a pro-inflammatory cytokine response including production of the IDO-inductor TNF-α, and has the potential to also influence neurotransmittor systems directly by crossing the blood brain barrier. We suggest that future studies should target the effects of DON on pig behavior and aim to document the relationship between behavioral changes and DON-related induction of proinflammatory cytokines.

### Amino Acid Composition of Feed

The inflammatory response changes amino acid metabolism. Firstly, protein synthesis is geared toward production of immunoglobulins and acute phase proteins. Secondly, as already discussed, cytokines will activate the enzyme IDO to catalyze the breakdown of tryptophan to kynurenine.

The altered amino acid metabolism may influence amino acid requirements, and therefore also change how diet composition influences behavior. Following experimental infection with Mycoplasma hyopneumoniae and swine influenza virus, post prandial concentrations of histidine, arginine, tyrosine and threonine were reduced (171). Acute phase proteins, such as fibrinogen, C-reactive protein and haptoglobin, which are synthesized in response to immune activation, have a higher content of tryptophan, tyrosine and phenylalanine when compared to muscle protein (172). Their synthesis in inflammatory states might therefore modify dietary requirements and induce metabolic deficiencies which stimulate exploratory oral behaviors in pigs. Diets deficient in tryptophan can indeed increase activity and attraction to blood (173), although another study did not find large effects on the attraction to blood after reducing daily protein availability (174). Pastorelliet al. (175) showed that pigs which were individually housed in poor sanitary conditions, inducing immune activation as evidenced by haptoglobin concentrations, showed decreased growth, and increased activity and trough-related exploratory behavior in response to a reduction in diet quality, whereas such an effect was not seen in good sanitary conditions. This might indicate a greater susceptibility to specific nutrient deficiency in immune-stimulated pigs, but the consequences for abnormal injurious behaviors toward penmates could not be evaluated in these individually housed animals. A recent study reported increased occurrence of damaging behaviors in pigs on a diet low in protein (176). Pigs fed a protein deficient diet, when subjected to both high or low sanitary conditions, showed increased ear biting, tail biting and other oral manipulation directed to penmates. Furthermore, the pigs subjected to low sanitary conditions, which led to increased lung problems, raised haptoglobin levels and other changes in immune status, showed increased damaging behavior directed at pen mates and damage to ears as compared with the pigs kept in high sanitary conditions (66). The effect of sanitary conditions on ear biting was diet dependent. Supplementing the diet with a 20% increase of amino acids considered to be important in the inflammatory response (i.e., tryptophan, threonine, and methionine) attenuated the effect of low sanitary conditions on ear biting. As poor sanitary conditions modify immune status (66), the interaction between sanitary conditions and diet on behavior may be an example of how several detrimental factors could interact through cytokine production and changes in amino acid metabolism to increase the likelihood of damaging behavior (see Synthesis).

### The Influence of Gut Microbiota on the Immune Response and on Neurotransmitter Balance

In 2016, Brunberg et al. highlighted the potential role of gut microbiota in the development of damaging behavior in poultry (feather pecking) and pigs (tail biting) (177). In this paragraph we will attempt to link this important work to the current hypothesis by briefly summarizing the ways in which gut microbiota can influence the immune response and neurotransmitter balance.

The gut microbiota refers to the commensal bacteria in the gut. They are necessary for the digestion and absorption of complex carbohydrates. The gut microbiota is established early in life, and is important for the maturation of the immune system and stress axis (reviewed by (178); for a description of porcine gut microbiota at different ages see (179)). The microbiota could therefore influence the pathways discussed in this review either by changing the set point of the immune system and HPA axis in the individual, or by interacting with the risk factors discussed here as they occur throughout the lifespan. Illness, unsanitary housing, psychological stress, mycotoxins in food, are all stressors, and as such may influence the microbiota. One consequence of this could be an increase in intestinal wall permeability, with endotoxins from bacteria entering the circulation and thereby worsening the inflammatory state. It has been suggested that the increased inflammatory state seen in depressed individuals (humans) is due to a response to LPS in intestinal bacteria that reach the circulation due to increased permeability in the intestinal wall (180, 181). Hens with high and low levels of repetitive feather-pecking behavior have differences in intestinal microbial metabolites (182), but transplantation of gut microbiota from the high to low feather pecker line and vice versa did not have a strong effect on microbiota nor on feather pecking (183). A recent report showed more lactobacilli in the gut microbiota of pigs that neither bit nor were bitten compared to biters and victims (Rhabi et al., under review).

Antibiotic treatment in response to pathogens (Pathogens and immune activation) could reduce microbiotal complexity [measured as number of different genera; see (178)]. It should be noted that research on germ-free (=microbiota-free) mice and rats have yielded different results regarding the anxiety of microbiota-free individuals. Germ-free mice display less anxiety-related behavior, while germ-free rats show increased anxiety. It is therefore prudent not to extrapolate the rapidly increasing literature on the effect of microbiotal manipulations in mice directly to pigs (178). One of the most interesting features of the microbiota in the perspective discussed here, is that certain bacterial genera can metabolize tryptophan, synthesize tryptophan and even (at least *in vitro*) produce serotonin (178). Thus, the composition of the microbiota may, in addition to diet composition, influence tryptophan availability.

In summary, there is no conflict between the gut-brain-axis as the physiologic system linking the different risk-factors for damaging behaviors in hens and pigs (177), and the ideas discussed in the current paper. Rather, both hypotheses highlight how important it is to look beyond single biomarkers to attempt to understand the lifetime impact of diet, environment, internal and external risk factors in shaping the social behavior of the individual. This will be disussed further in the synthesis (5).

## Synthesis

We have summarized evidence that immune activation can influence mood in a negative way, possibly causing both lethargy, hopelessness, sadness, anxiety and increased irritability. We have discussed how different risk factors for tail biting in pigs, both infectious (Pathogens and immune activation) and non-infectious (The housing environment and immune activation, Psychological stress and immune activation, Mycotoxins in feed), activate the immune system, and how the outcome of exposure to any risk factor may depend on the nutritional status of the pigs, and the composition, quality and availability of feed (Amino acid composition of feed). Importantly, though the focus of this paper is on mood changes caused by cytokines, it does not exclude other explanations such as damaging behavior being induced by the pigs trying to compensate for depletion of nutrients due to inflammatory processes by redirecting foraging behavior toward penmates. Both of these processes (cytokine dependent and independent) may also run in parallel.

From field experience, anecdotes and also from epidemiological reports, there are strong indications that the effects of risk factors are cumulative, and that this explains why one risk factor may change nothing in one farm, but be the proverbial drop that makes the cup run over and causes a tail biting outbreak in another. The hypothesis that the risk factors work through immune activation gives a framework for understanding how they add up on a mechanistic level. While this is quite easy to envisage when the risk factors have a close temporal relationship, it turns out that the innate immune system also can remember earlier insults and be shaped by them. This phenomenon where the innate immune system changes its response to a stimulus depending on previous history is called innate immune memory (IMM). Adult rats who had been experimentally infected with *E. coli* at 4 days after birth had a much stronger increase in pro-inflammatory cytokines in the CNS in the response to LPS injection at 2–3 months of age than the non-infected controls (184). When testing effects of this “two-hit” paradigm on cognitive ability, the researchers found that rats that only experienced the experimental infection did not show any impairment unless they were also exposed to LPS as adults (185). This is probably linked to microglial activation. Microglia, the resident macrophages of the brain, are normally quiescent in healthy individuals. However, through exposure to pathogens in early life, the microglia may change from active to over-active when the individuals are exposed to a second challenge as adults [*ibid* and see also Neher and Cunningham (186)]. Thus, it is conceivable that poor health in weaners could shape the response to e.g., mycotoxin-contaminated food or psychological stress due to mixing in the finisher phase, leading to a stronger response in the individuals who have a previous history of poor health, and that microglia activation could be central to the observed changes in physiology and behavior. Microglia of mammals can become quite old [several years in humans, see Reu et al. (187)], possibly “remembering” stimuli over a long time-span, and while microglial priming has mostly been discussed in the context of neurological disease in humans, it could also be involved in more low-key changes in social motivation and change the threshold for showing damaging behavior toward conspecifics. While more studies are needed to test this hypothesis, we believe that by interpreting negative events in the life of a pig, whether it be in the form of social stress, illness or contaminated feed, in the context of what these challenges do to the immune system, we will develop a more complete picture of how health and behavior are interrelated.

Understanding microglial priming in the context of pig health and welfare is an important research goal, including understanding whether some individuals are more susceptible to long-lasting neuroinflammation than others, and if so, why. Another important direction for future research is to design studies that test for causal relationships. In an experiment using LPS as immune-stimulating agent, pigs that were injected with LPS showed a shift in social motivation and performed more tail- and ear- directed behavior than saline-injected pigs at 2 days after injection (82), indicating that the inflammatory response caused the change in phenotype. However, one disadvantage of LPS is that it lacks the slower onset characteristic of some naturally occurring diseases as well as environmental risk factors and might not give us a complete picture of how the immune system influences behavior, even though it has been important in identifying candidate mechanisms and biomarkers. As mentioned earlier, alongside with testing for causality, a complete understanding of the pathways from cytokines to altered behavior is lacking. One important challenge is that most data on mechanisms are derived, of necessity, from physiological and behavioral snapshots where animals are sacrificed shortly after treatment, and brain tissue analyzed for selected biomarkers. Therefore, the results will depend on the time-point of analysis (in addition to the selected brain areas that are sampled), and these snap-shots do not give a full understanding of the chain of events that may ultimately lead to a change in the threshold for inflicting damage on penmates. Plasma biomarkers known to be associated with changes in brain metabolism or signaling pathways, together with more sensitive measures of behavioral changes, could be an approach to overcome the “one time-point” analysis and also improve our understanding of the dynamics behind immune-driven changes in behavior over a much longer time perspective than the (maybe not biologically relevant) 24 or 48 h commonly used. Lastly, by studying not only damaging behavior but also more subtle positive social interactions, we could increase our understanding of how immune activation influences the individual's function within a group as well as this individual's welfare.

## Data Availability Statement

The original contributions presented in the study are included in the article/supplementary material, further inquiries can be directed to the corresponding author/s.

## Author Contributions

All authors listed have made a substantial, direct and intellectual contribution to the work, and approved it for publication.

## Conflict of Interest

The authors declare that the research was conducted in the absence of any commercial or financial relationships that could be construed as a potential conflict of interest. The handling editor declared a past co-authorship with the authors SE and ID.
